# A collection of three-dimensional datasets of hydrating cement paste

**DOI:** 10.1016/j.dib.2023.108903

**Published:** 2023-01-13

**Authors:** Michal Hlobil, Ivana Kumpová

**Affiliations:** a Institute for Building Materials, ETH Zurich, Switzerland; b Institute of Theoretical and Applied Mechanics of the Czech Academy of Sciences, Prague, Czech Republic

**Keywords:** Image processing, Microstructure characterization, Portland cement paste, Specific surface area, X-ray micro-computed tomography

## Abstract

This dataset contains a collection of digitized three-dimensional hardened cement paste microstructures obtained from X-ray micro-computed tomography. Four sets of ordinary Portland cement-based pastes were produced and X-ray screened, varying in the initial water-to-cement ratio (wcr=0.35 and 0.50) and fineness of cement used (391 and 273 m^2^/kg Blaine). Individual paste samples from each set were screened after 1, 2, 3, 4, 7, 14, and 28 days of elapsed hydration at 20˚C in saturated conditions. Each digitized paste specimen captures a realistic spatial configuration of the principal microstructural phases (anhydrous cement, hydration products, and large capillary porosity). The dataset may be further used for assessing changes in the mix design on the resultant spatial configuration of the paste microstructure or aid the development of microstructure-inspired micromechanical models based on realistic material configuration.

**Specifications Table**

**Table:** 

Subject	Computational Materials Science
Specific subject area	Evolution of Portland cement paste microstructure
Type of data	Image Figure Table
How the data were acquired	X-ray computed tomography screening of specimen at designated age using a twinned orthogonal adjustable tomograph TORATOM with an in-house developed testing protocol; 3D image reconstruction from a stack of 2D projections using VG Studio Max 3.4 software with digital image correlation applied; image filtering using Fiji 2.9.0 (ImageJ library) with “xlib” plugin to remove imaging artifacts.
Data format	Raw Extracted Filtered
Description of data collection	Four sets of hardened Portland cement pastes were prepared, varying in the initial water-to-cement ratio (wcr=0.35 and 0.50) and fineness of cement used (391 and 273 m^2^/kg Blaine). Paste samples from each set were screened after approx. 1, 2, 3, 4, 7, 14, and 28 days of elapsed hydration at 20˚C in saturated conditions.
Data source location	• Institution: Institute of Theoretical and Applied Mechanics of the Czech Academy of Sciences • City: Prague • Country: Czech Republic
Data accessibility	Repository name: Zenodo Data identification number: 10.5281/zenodo.7275143, 10.5281/zenodo.7275149, 10.5281/zenodo.7275158, 10.5281/zenodo.7275174 Direct URL to data: , 10.5281/zenodo.7275149, 10.5281/zenodo.7275158, 10.5281/zenodo.7275174
Related research article	M. Hlobil, I. Kumpová, A. Hlobilová, Surface area and size distribution of cement particles in hydrating paste as indicators for the conceptualization of a cement paste representative volume element, Cem Concr Compos 134 (2022) 104798. 10.1016/j.cemconcomp.2022.104798

## Value of the Data

• The three-dimensional datasets capture a realistic spatial configuration of the principal microstructural phases in hardened cement paste (anhydrous cement, hydration products, and large capillary pores) and their evolution throughout hydration as a function of the initial water-to-cement ratio and cement fineness.
• This dataset allows to assess the changes in the mix design on the resultant hardened cement paste microstructure, which is useful for material scientists to deepen the understanding of the mix design parameters.
• Researchers relying on microstructure-inspired micromechanical models can use the three-dimensional images with realistic microstructures instead of artificially-developed microstructural models to directly perform numerical simulations.
• A quantitative or statistical characterization of the principal microstructural phases may be carried out, e.g. studying the role of a cement particle size distribution or specific surface area development throughout hydration [1], or the three-dimensional images may be compared to artificially-generated microstructural models to assess their accuracy.

## 1 Objective

This dataset provides a concise collection of three-dimensional images capturing microstructural evolution in hydrating cement paste, minimizing the possible scatter and incompatibility which arise when collecting data from various sources. All specimens underwent identical preparation, treatment and measurement protocol, which was fully adapted to the dimensions of the specimens and material tested, and aimed at providing the largest imaging resolution of the material microstructure. The digitized three-dimensional images capture microstructural details in a cylindrical paste specimen with a macroscopic diameter of ∼1 mm with an accuracy (imaging resolution) with a voxel size of ∼1.1 µm.

## 2 Data Description

The dataset [2], [3], [4], [5] contains a collection of digitized three-dimensional hardened cement paste microstructures obtained from X-ray micro-computed tomography. It captures their evolution in time, from an early age (termed hereafter ∼1 d of elapsed hydration) to maturity (after ∼28 days) at defined curing conditions. Varying the initial water-to-cement mass ratio (wcr=0.35 and 0.50) and using one type of cement with a variable fineness (391 and 273 m^2^/kg Blaine) but otherwise identical in mineralogical composition yielded four distinct pastes with a unique spatial configuration of the microstructure.

All specimens from each paste type are stored in a separate folder with a unique filename. Each specimen was X-ray screened at a designated time corresponding to an age of 1, 2, 3, 4, 7, 14, and 28 days of elapsed hydration at 20˚C in saturated conditions. A unique specimen was used for each X-ray screening to avoid the possible accumulation of microstructural defects within one specimen, originating from specimen manipulation or sequential measurement. Thus, seven distinct specimens for each paste type were X-ray screened at given times. Consequently, the digitized microstructures do not show the evolution of a single specimen in time, but rather a generalized evolution of the microstructure of a given paste type.

The three-dimensional digitized microstructures are saved as uncompressed and unprocessed *.tif (raw) greyscale image data files in 16-bit image depth (as unsigned integers) in a little-endian byte sequence. Each raw file has a size of ∼2.7 GB and contains a cylindrical specimen embedded into a cubic volume with a side length of 1100 voxels (=3D pixels) and a variable voxel size in the range 1.0913 − 1.1174 µm depending on the particular specimen, as specified in the file name.

A smaller image subvolume, denoted as Region Of Interest (ROI), was extracted from the interior of the full-sized specimen to facilitate processing and mutual microstructural comparison. This ROI has a cubic shape and a fixed size of 500 × 500 × 500 µm^3^ constituted by a three-dimensional voxel matrix with variable count (as per the actual voxel size). Both the voxel count and size are further specified in the file name. Each ROI is also saved as a 16-bit little-endian *.tif (raw) file occupying ∼180 MB per digitized microstructure.

**Fig. 1:**
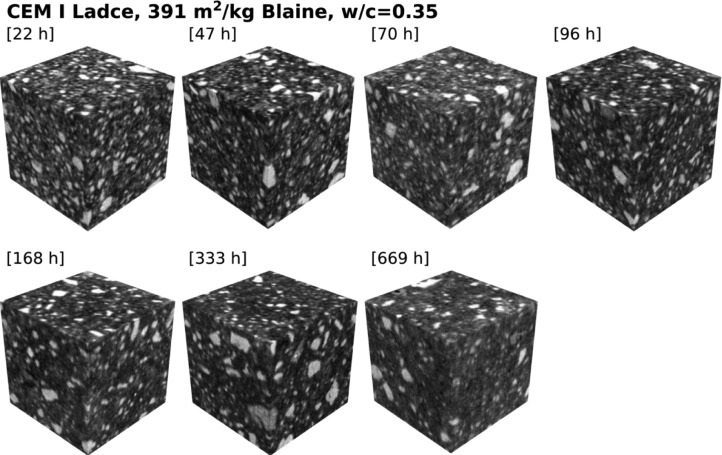
Digitized cement paste microstructures made from CEM I Ladce (391 m^2^/kg Blaine) and water-to-cement ratio of 0.35; raw data available from [2].

**Fig. 2:**
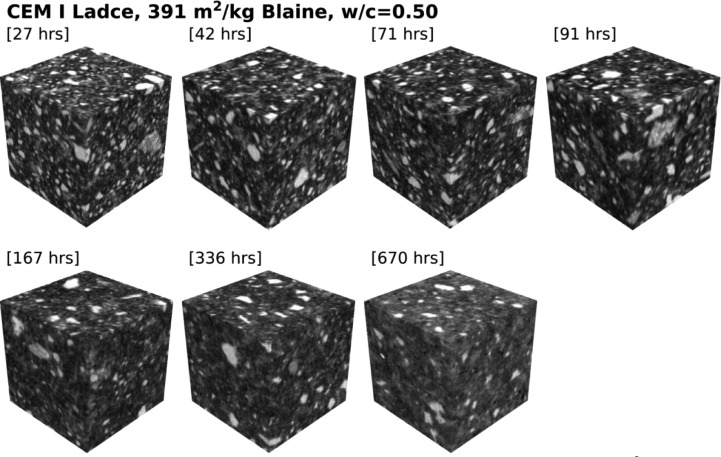
Digitized cement paste microstructures made from CEM I Ladce (391 m^2^/kg Blaine) and water-to-cement ratio of 0.50; raw data available from [3].

**Fig. 3:**
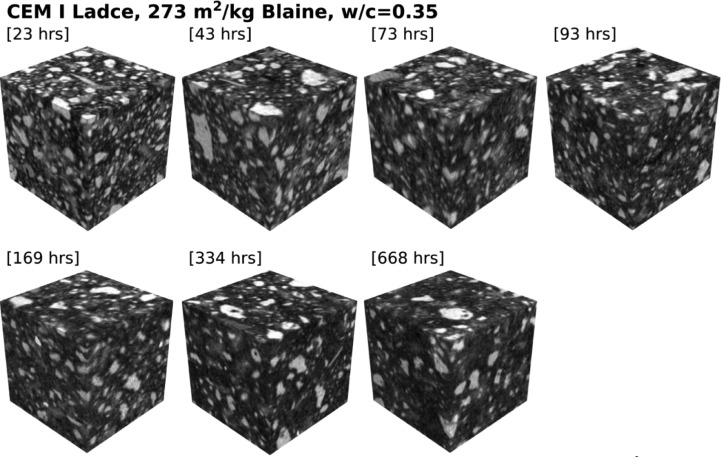
Digitized cement paste microstructures made from CEM I Ladce (273 m^2^/kg Blaine) and water-to-cement ratio of 0.35; raw data available from [4].

**Fig. 4:**
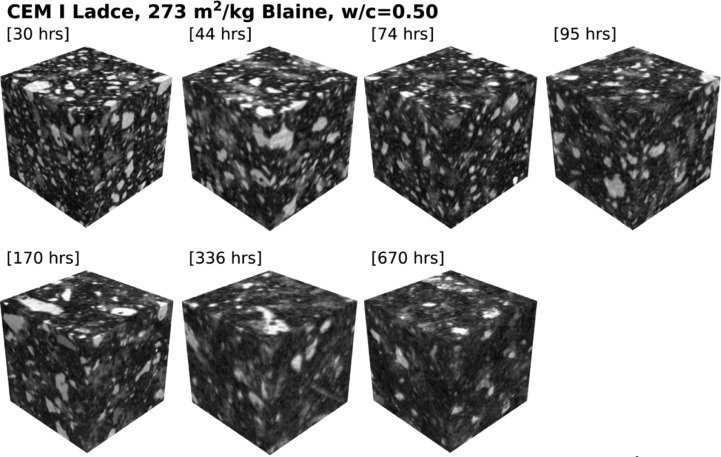
Digitized cement paste microstructures made from CEM I Ladce (273 m^2^/kg Blaine) and water-to-cement ratio of 0.50; raw data available from [5].

A gallery of ROIs is shown in Fig. 1, Fig. 2, Fig. 3, Fig. 4 for each water-to-cement ratio and cement fineness used, and further specification is reported in Tables 1, 2, 3, 4, respectively. Note that while the full specimens (with a file name starting as CEM-I-Ladce_*) are provided “directly as obtained” from the 3D reconstruction algorithm and are further kept unmodified and unprocessed, the ROIs (with a file name starting as filteredROI_*) were first extracted from the bulk volume and then additionally post-processed by applying several filters to increase the imaging contrast of phases, refer to the next Section for details.

**Table 1:** Specification of dataset for cement paste made from CEM I Ladce (391 m^2^/kg Blaine) and water-to-cement ratio of 0.35.

Specimen age [hrs]	ROI size [voxels]	Voxel size [µm]
22	455 × 455 × 455	1.0991
47	451 × 451 × 451	1.10913
70	451 × 451 × 451	1.10935
96	455 × 455 × 455	1.0991
168	455 × 455 × 455	1.0991
333	455 × 455 × 455	1.0987
669	451 × 451 × 451	1.10948

**Table 2:** Specification of dataset for cement paste made from CEM I Ladce (391 m^2^/kg Blaine) and water-to-cement ratio of 0.50.

Specimen age [hrs]	ROI size [voxels]	Voxel size [µm]
27	448 × 448 × 448	1.1174
42	455 × 455 × 455	1.0991
71	451 × 451 × 451	1.10935
91	455 × 455 × 455	1.099
167	451 × 451 × 451	1.10922
336	451 × 451 × 451	1.11
670	451 × 451 × 451	1.10948

**Table 3:** Specification of dataset for cement paste made from CEM I Ladce (273 m^2^/kg Blaine) and water-to-cement ratio of 0.35.

Specimen age [hrs]	ROI size [voxels]	Voxel size [µm]
23	455 × 455 × 455	1.0991
43	455 × 455 × 455	1.0991
73	451 × 451 × 451	1.10935
93	455 × 455 × 455	1.099
169	451 × 451 × 451	1.10922
334	455 × 455 × 455	1.0987
668	451 × 451 × 451	1.10952

**Table 4:** Specification of dataset for cement paste made from CEM I Ladce (273 m^2^/kg Blaine) and water-to-cement ratio of 0.50.

Specimen age [hrs]	ROI size [voxels]	Voxel size [µm]
30	448 × 448 × 448	1.1174
44	455 × 455 × 455	1.0991
74	451 × 451 × 451	1.10935
95	455 × 455 × 455	1.099
170	451 × 451 × 451	1.10922
336	455 × 455 × 455	1.0987
670	451 × 451 × 451	1.10952

To summarize the employed file naming convention used herein, we use the file “CEM-I-Ladce_Blaine391_wcr035_t168hrs_1100×1100×1100voxels_1d0991um_LittleEndian_UInt16.tif” from [2] as an example. Herein, the prefix “CEM-I-Ladce_” denotes the full-sized specimen (i.e. a cube with a side length of 1100 voxels), “Blaine391” the fineness of cement used (i.e. the Blaine specific surface area amounting herein to 391 m^2^/kg), “wcr035” the initial water-to-cement mass ratio used for the paste (here w/c=0.35 [-]), “t168hrs” the specimen age during X-ray screening (here after 168 hrs of elapsed hydration), “1100×1100×1100 voxels” the voxel matrix dimensions (as previously signaled by the file prefix CEM-I-Ladce_), “1d0991um” the voxel size (here 1.0991 µm/voxel), concluded by “LittleEndian_UInt16.tif” denoting the byte order, data format, and image format of the file. File names starting with “filteredROI_” denote cropped and extracted cubic ROIs with smaller and variable voxel matrix dimensions; however, all such specimens provided herein feature a constant 500 µm side length (and thus variable voxel count).

The first two datasets [2,3] consist of digitized cement paste microstructures made from a finely-ground Portland cement classified as CEM I [6], with a variable water-to-cement ratios amounting to 0.35 and 0.5, respectively, see Figs. 1 and 2 for an overview of ROIs.

The second two datasets [4,5] contain hardened paste data made from coarsely-ground cement featuring identical wcr as was used in the former case. While this cement shares an identical mineralogical composition, it differs from the former in its fineness and, consequently, in its particle size distribution, see Figs. 3 and 4 for a qualitative difference.

## 3 Experimental Design, Materials and Methods

Before specimen production, the raw materials were conditioned to laboratory temperature (20±1˚C). The anhydrous cement was mixed with distilled water in a laboratory mixer (Waring blender, USA) using 3000 RPM for 3 minutes. The continuous mixing was interrupted every 60 s to scrape any accumulated unmixed cement from the inner wall of the mixing bowl to ensure a homogeneous paste. The fresh slurry was manually loaded into a plastic syringe and pressure-injected into a plastic pipette tip. Its internal dimensions and, thus, the resultant cylindrical specimen size amounted to a diameter of ∼1 mm and length (height) of ∼5 mm. To minimize the water evaporation from the fresh slurry, both ends of the pipette tip were sealed with a paraffin wax foil, and the specimen was placed in a water bath to maintain saturated conditions and a stable temperature of 20˚C during hardening. Note that the specimens were sequentially not demoulded prior to X-ray micro-computed tomography (XµCT) measurements as the plastic tip is effectively transparent to the X-rays, and further specimen manipulation could have damaged the brittle specimen.

The screening of hardened cement paste was carried out on a patented [7] twinned orthogonal adjustable cone-beam tomograph used for X-ray micro-tomography measurements, positioned atop an anti-vibration platform with active air damping. The test setup consisted of an X-ray tube XWT-160-TCHR (X-ray WorX, Germany) operating in nanocofus mode with a power of 1.8 W, acceleration voltage of 60 kV, and target current of 30 µA. Such setup ensured a spot size of approx. 1 µm, which was the target for the measurement, as well as the top limit in accuracy for the test setup used. The X-rays passing through the sample were captured by a flat panel detector Dexela 1512NDT (PerkinElmer inc., USA), featuring a native pixel size of 74.8 µm in a matrix of 1536 × 1944 pixels. The specimen was positioned between the X-ray source and the detector to archive a projection magnification ratio of 67 − 68x and a projection pixel size of ∼1.1 µm. During the screening, the specimen was axially rotated and stopped in predefined 1440 equiangular positions, where the 2D projections were recorded, each with an exposure time of 2.5 s.

The image reconstruction was carried out using a CT reconstruction module from VG Studio Max 3.4 software (Volume Graphics, Germany) using an iterative reconstruction method (algebraic reconstruction technique, ART). Prior to the reconstruction of the 3D image, the 2D projections were filtered to compensate for any defective pixels present on the detector and to remove uneven exposure and possible beam-hardening image defects. In addition to the applied filters, any ring artifacts stemming from the axial specimen rotation were removed during the reconstruction process. As the hardened cement paste specimen shape was cylindrical, a circumscribed cube with a side length of 1100 voxels was created and exported for further processing.

A Region of Interest (ROI) was sequentially extracted from the full reconstructed volume. Its size was fixed to 500 × 500 × 500 µm^3^ but contained a variable voxel count, see Table 1, Table 2, Table 3, Table 4. These ROIs were further filtered to enhance imaging contrast using the ImageJ/Fiji tool kit, particularly the “xlib” plugin. Two imaging filters from “xlib” were used, first the “Remove background” to compensate for uneven exposure, followed by “Anisotropic diffusion” [8] to sharpen the contrast among the different microstructural constituents and blur areas with low-intensity gradient. The image histogram of each ROI was normalized by contrast stretching prior to each filter applied.

## Ethics Statements

The authors followed universally expected standards for ethical behavior in conducting and publishing scientific research. The work presented here did not involve use of human subjects or animal experiments. Also, the data was not collected from social media platforms.

## CRediT authorship contribution statement

**Michal Hlobil:** Conceptualization, Methodology, Investigation, Formal analysis, Resources, Data curation, Writing – original draft, Writing – review & editing, Visualization, Supervision, Project administration, Funding acquisition. **Ivana Kumpová:** Data curation, Methodology, Formal analysis, Investigation.

## Declaration of Competing Interest

The authors declare that they have no known competing financial interests or personal relationships that could have appeared to influence the work reported in this paper.

## Data Availability

Three-dimensional dataset of hydrating cement paste (CEM I Ladce, 391 m^2/kg Blaine, w/c=0.35) in TIFF format (Original data) (Zenodo.org). Three-dimensional dataset of hydrating cement paste (CEM I Ladce, 391 m^2/kg Blaine, w/c=0.50) in TIFF format (Original data) (Zenodo.org). Three-dimensional dataset of hydrating cement paste (CEM I Ladce, 273 m^2/kg Blaine, w/c=0.35) in TIFF format (Original data) (Zenodo.org). Three-dimensional dataset of hydrating cement paste (CEM I Ladce, 273 m^2/kg Blaine, w/c=0.50) in TIFF format (Original data) (Zenodo.org).

## References

[1] Hlobil M., Kumpová I., Hlobilová A. (2022). Surface area and size distribution of cement particles in hydrating paste as indicators for the conceptualization of a cement paste representative volume element. Cem. Concr. Compos..

[2] M. Hlobil and I. Kumpová, Three-dimensional dataset of hydrating cement paste (CEM I Ladce, 391 m2/kg Blaine, w/c=0.35) in TIFF format, Zenodo.org, v2, 2022. doi:10.5281/zenodo.7275143.

[3] M. Hlobil and I. Kumpová, Three-dimensional dataset of hydrating cement paste (CEM I Ladce, 391 m2/kg Blaine, w/c=0.50) in TIFF format, Zenodo.org, v2, 2022. doi:10.5281/zenodo.7275149.

[4] M. Hlobil and I. Kumpová, Three-dimensional dataset of hydrating cement paste (CEM I Ladce, 273 m2/kg Blaine, w/c=0.35) in TIFF format, Zenodo.org, v2, 2022. doi:10.5281/zenodo.7275158.

[5] M. Hlobil and I. Kumpová, Three-dimensional dataset of hydrating cement paste (CEM I Ladce, 273 m2/kg Blaine, w/c=0.50) in TIFF format, Zenodo.org, v2, 2022. doi:10.5281/zenodo.7275174.

[6] (2000). EN 197-1: cement – Part 1: composition, specifications and conformity criteria for common cements. Tech. Rep..

[7] T. Fíla, D. Vavřík, A multi-axial apparatus for carrying out x-ray measurements, particularly computed tomography, EU patent No. 2835631 (11. February 2015)

[8] Münch B. (2021). https://imagej.net/plugins/xlib/.

